# A 9-Year Teledermoscopy Service in New Zealand: Retrospective Service Review

**DOI:** 10.2196/36351

**Published:** 2022-10-06

**Authors:** Novell Shu Chyng Teoh, Amanda Oakley

**Affiliations:** 1 Te Whatu Ora Waikato Hamilton New Zealand; 2 Waikato Clinical Campus The University of Auckland Hamilton New Zealand

**Keywords:** dermatology, dermoscopy, telemedicine, skin neoplasms, melanoma

## Abstract

**Background:**

A teledermoscopy service was established in January 2010 wherein patients attended nurse-led clinics for the imaging of lesions of concern and remote diagnosis by a dermatologist.

**Objective:**

This study aims to review the number of visits, patient characteristics, the efficiency of the service, and the diagnoses made.

**Methods:**

We evaluated the waiting times and diagnoses of skin lesions for all patient visits from January 1, 2010, to May 31, 2019. The relationships between patient characteristics and the diagnosis of melanoma were specifically analyzed.

**Results:**

The teledermoscopy clinic was attended by 6479 patients for 11,005 skin lesions on 8805 occasions. Statistically significant risk factors for the diagnosis of melanoma and melanoma in situ were male sex (*P*<.001), European ethnicity (*P*=.001), an age of 65 to 74 years (*P*=.001), and Fitzpatrick skin type 2 (*P*=.001). Attendance was maximal during 2015 and 2016. The seasonal variations in visits from 2011 to 2018 revealed a consistent peak at the end of summer and a dip at the end of winter. In the year 2010, a total of 306 patients attended the clinic; 76.1% (233/306) of these patients were discharged to primary care, and 23.9% (73/306) were referred to a hospital for a specialist assessment. For patients who were diagnosed with suspected melanoma by a dermatologist from January 1, 2010, to May 31, 2019, the median waiting time for an imaging appointment was 44.5 (mean 57.9; range 8-218) days. The most common lesions diagnosed were benign naevus (2933/11,005, 26.7%), benign keratosis (2576/11,005, 23.4%), and keratinocytic cancer (1707/11,005, 15.5%); melanoma was suspected in 4.6% (507/11,005) of referred lesions. The positive predictive value of melanoma and melanoma in situ was 61.1% (320 true positives and 203 false positives). The number needed to treat (ie, the ratio of the total number of excisions to the number with a histological diagnosis of melanoma or melanoma in situ) was 2.02.

**Conclusions:**

A teledermoscopy service offered by nurse-led imaging clinics can provide efficient and convenient access to dermatology services by streamlining referrals to secondary care and prioritizing patients with skin cancer for treatment.

## Introduction

New Zealand had the second highest rate of melanoma worldwide in 2018, following Australia [1]. In 2017, the New Zealand Cancer registry recorded 2553 cases of melanoma, with an age-standardized incidence rate of 35.1 per 100,000 people, and melanoma was one of the top 10 causes of cancer death among both women and men in 2016, 2017, and 2018 [2]. It was predicted that 90,400 New Zealanders would be diagnosed with at least one in situ or invasive keratinocytic cancer (also known as *nonmelanoma skin cancer*) in 2018 [3]. Diagnostic uncertainty results in high rates of referrals to dermatologists. Access to dermatology outpatient clinics in New Zealand is limited by a shortage of dermatologists [4,5], resulting in unnecessary excisions of benign lesions in primary care and, potentially, the late diagnosis of melanoma [6]. The New Zealand Ministry of Health’s Faster Cancer Treatment targets include a 2-week indicator for ensuring that patients with a high suspicion of cancer are seen by a specialist service within 2 weeks of being referred; however, these targets have been difficult for district health boards to achieve. For patients with a low suspicion of cancer, the expected waiting time is 45 days for a semiurgent outpatient clinic appointment and 120 days for a routine outpatient clinic appointment [7].

There has been rapid growth in the use of store-and-forward teledermoscopy globally, and it has proven to be a valuable service for both clinicians and patients worldwide. A 14-year study of UK teledermatology services showed 68% diagnostic concordance and an 82% satisfaction rate for 40,201 teleconsultations [8]. Another study in Spain showed improved access to dermatologists through a teledermoscopy service [9]. The use of teledermoscopy has allowed skin lesions to be diagnosed at remote locations and has reduced the need for face-to-face consultations. Following a proof-of-concept study that was conducted in 2008 to confirm whether skin lesions could be diagnosed from high-quality digital photographs [10], a teledermoscopy service was established at our center in January 2010. This teledermoscopy service is a collaboration between a public hospital and an established private teledermoscopy company. In an earlier trial of 200 patients who used a similar service that was provided at another center in New Zealand, the service resulted in potential financial savings and shorter waiting times [11].

Through our service, patients referred from primary care for the assessment of 1 to 5 skin lesions may be scheduled for an appointment at an imaging clinic in 1 of 3 towns. Information, including digital images, is collected at these clinics. A specially trained nurse (ie, a melanographer) collects demographic and medical information and captures regional, close-up, and dermoscopic images of the skin lesions that were identified in the referral for later remote diagnosis by a teledermatologist. Additional skin lesions of patient or nurse concern can also be imaged. Total body skin examinations were not offered during the time covered by this study.

This study aims to record the number of visits over a 9-year and 7-month period, patient characteristics, the efficiency of the service, and the diagnoses made.

## Methods

### Ethical Considerations

New Zealand Health and Disability Ethics Committee approval was not required for this research, as it was a low-risk retrospective service review.

### Recruitment

All patient visits to the teledermoscopy clinic from January 1, 2010, until May 31, 2019, were included in this study. We also performed analyses on subsets of patients (ie, patients with confirmed melanoma and patients who attended the clinic from January 2010 until December 2010).

Regional images were captured by using a Nikon D3300 (Sendai Nikon Corporation), and macroscopic and dermoscopic images were captured by using a DermLite Cam v01 (3Gen LLC) and DermLite Cam v02 (3Gen LLC); other cameras were used in the first few years. Files were uploaded by using a virtual private network for storage on a secure server. The files were downloaded remotely to be viewed, using proprietary software. Each case was assessed by a dermatologist, who made a diagnosis and formulated a management plan. The referring primary care physicians and patients expected to receive a diagnosis report within 7 to 10 working days after the patients’ appointments.

The dates of imaging, patient-related data, and lesion-specific data were extracted from the service database. The recorded demographic information (age, sex, and ethnicity) and data on melanoma risk factors, such as Fitzpatrick skin type (1: pale, burns easily; 2: fair, burns easily; 3: darker white, tans easily; 4: brown, tans easily; 5-6: dark brown or black, always tans), eye and hair color, a personal and family history of melanoma, outdoor occupation, and a history of sunburn, were collected. The diagnosis software was accessed to review the dates of referrals, patients’ medical and lesion histories, and skin lesion assessments. Patients’ hospital electronic health records were accessed to obtain histopathology results following the excision of suspected melanoma.

### Statistical Analysis

#### Risk Factors for Melanoma

Patient characteristics and risk factors were analyzed for a subset of patients with confirmed melanoma. Statistical tests (*Z* test, Pearson chi-square test, and Fisher exact test) were conducted to determine the significance of patient characteristics and the occurrence of melanoma.

A *Z* test was used to compare the statistical significance of the relationship between sex and the occurrence of melanoma. A Pearson chi-square test was used to compare the relationships among age, ethnicity, skin type, risk factors, and the occurrence of melanoma for sample sizes of <5, and a Fisher exact test was used to calculate the *P* value. Patients with “unsure” responses were excluded from the analysis.

#### Trend and Timeline

The number of visits over 2010 to 2018 and the number of visits for all months over this 9-year period were calculated. Visits in the year 2019 were excluded, as the data were only collected up to May 31, 2019. The number of patients with confirmed melanoma (in situ and invasive) were compared with the number of patient visits each month. A linear regression model was used to analyze the relationships among months, the number of visits, and the occurrence of melanoma over the 9-year period. The *R*^2^ value was calculated to predict how well the data fit the regression model.

#### Efficiency of the Service

We analyzed a subset of patients who were seen in the clinic from January until December 2010. The percentage of patients who were referred to a hospital for a specialist assessment was calculated. The waiting time for an appointment was based on the difference between the date of the referral and the date of imaging. Wait times for treatments (excision or discharge) were determined for a subset of patients.

#### Diagnoses

Skin lesions were classified via teledermoscopic diagnosis. Skin cancers were compared to the total number of skin lesions ([melanoma + keratinocytic cancer]/total lesions × 100). The percentages of benign and premalignant lesions were calculated. Based on the subset of skin lesions for which excision was recommended, the percentage of confirmed melanoma or melanoma in situ (based on histology), the number needed to treat (NNT), and positive predictive value (PPV) were also determined.

#### Quality Standards

Aspects of the teledermoscopy service were compared with the 2011 Quality Standards for Teledermatology by the British Association of Dermatologists [12].

## Results

### Recruitment

Between January 1, 2010, and May 31, 2019, a total of 6479 patients attended the teledermoscopy clinic on 8805 occasions (female: 4087/6479, 63.1%; male: 2392/6479, 36.9%). The majority of visits were of physician concern (5608/8805, 63.7%), and the remainder (3202/8805, 36.3%) were referred due to patients’ concerns. Images were taken of 11,005 unique skin lesions.

The median age of the 6479 patients was 57 years (mean 53.67 years; range 2 months to 100 years). As per Table 1, most patients self-identified as New Zealand European (5800/6479, 89.5%). The remaining patients self-identified as Maori (321/6479, 5%), Pacific Islander (29/6479, 0.4%), Asian (158/6479, 2.4%), and other (160/6479, 2.5%).

Among the 6479 patients, the Fitzpatrick skin type was recorded as type 1 for 437 (6.7%) patients, type 2 for 4022 (62.1%) patients, type 3 for 1478 (22.8%) patients, type 4 for 503 (7.8%) patients, and type 5 or 6 for 25 (0.4%) patients. The skin type of 14 (0.2%) patients were not recorded.

**Table 1:** Demographic characteristics of patients who attended the teledermoscopy service.

Demographic characteristics		All patients (N=6479)	Patients with histologically confirmed melanoma (n=330)
Age (years), median		57	68
Number of male patients:number of female patients (ratio)		2392:4087 (0.59)	176:154 (1.14)
**Ethnicity, n (%)**			
	European	5800 (89.5)	330 (100)
	Māori or Pacific Islander	350 (5.4)	0 (0)
	Asian	158 (2.4)	0 (0)
	Other^a^	160 (2.5)	0 (0)

^a^Mixed, Mediterranean, other White, and other Black patients.

### Risk Factors for Melanoma

The median age of 330 patients with histologically confirmed melanoma was 68 (mean 73.5; range 20-93) years. There was a statistically significant higher incidence of melanoma in men (176/330, 53.3%; SD 0.0175; *P*<.001). The 65 to 74 years age group had the highest occurrence of histopathologically confirmed melanoma (*P*=.001); all self-identified as European.

### Trend and Timeline

Clinic visit numbers were maximal in the years 2015 and 2016. The seasonal variations in visits from 2011 to 2018 revealed a consistent peak between March and April—the end of summer in New Zealand—and a dip between August and September—the end of winter. A linear regression model was used to demonstrate a statistically significant linear relationship among the variables (*P*<.001).

The lowest proportion of histologically confirmed melanomas was found in winter (72/1969, 3.65%; from June to August), and the highest was 4.37% (91/2082) in spring (from September to November). However, the *R*^2^ value was 0.22 when including outliers and 0.3 when not including outliers; hence, it is a poor sign that the predictive models and chi-square distribution test showed no statistically significant relationship between the months and the occurrence of melanoma (*P*=.65).

### Efficiency of the Service

**Figure 1:**
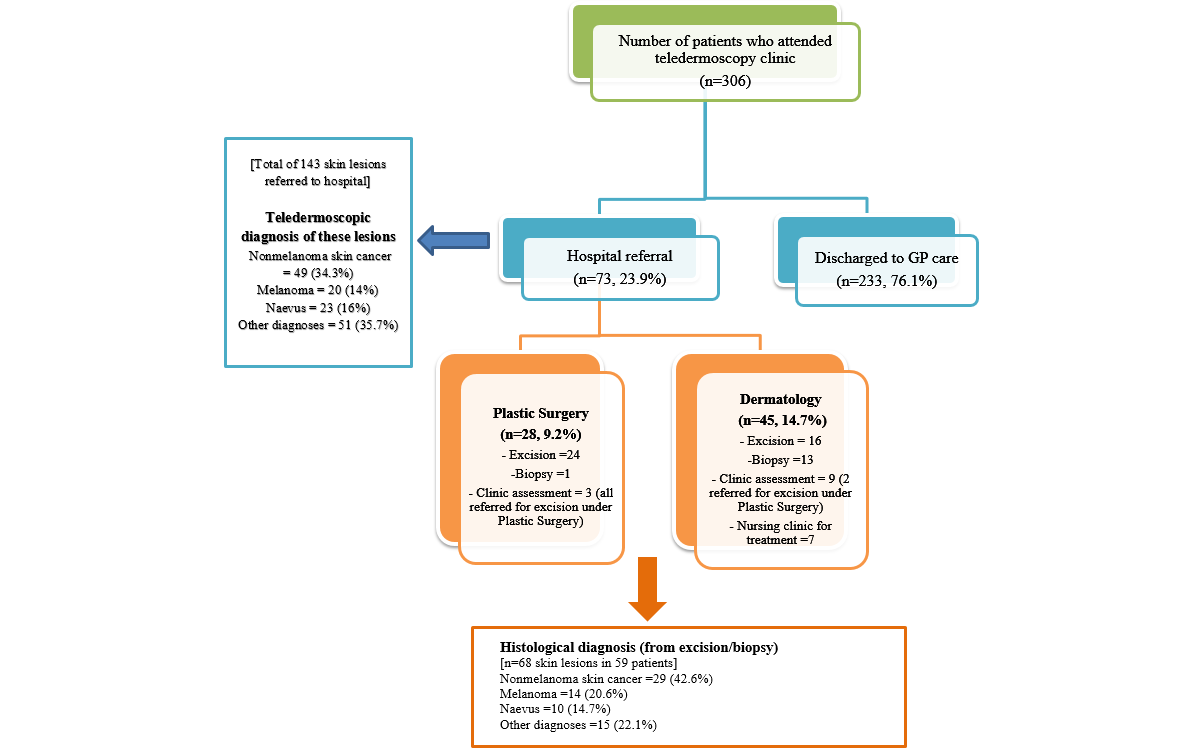
Flowchart of patients who attended the teledermoscopy clinic from January to December 2010. Other diagnoses included benign keratosis, vascular lesions, inflammatory lesions, collisions, other benign lesions, dermatofibroma, and uncertain diagnoses. GP: general practitioner.

Figure 1 shows the flowchart of patients who attended the teledermoscopy clinic from January to December 2010. Of the 306 patients seen during this period, 23.9% (n=73) subsequently required a hospital specialist appointment. Biopsy or excision was recommended for 59 (19.2%) patients with 68 lesions. In total, 76.1% (n=233) of patients were discharged back to primary care.

Between January 2010 and May 2019, melanoma was strongly suspected in 463 patients; they had a median waiting time of 44.5 (mean 57.9; range 8-218) days for imaging and a median waiting time of 63 (mean 63.2; range 28-94) for the first treatment received.

### Diagnoses

The teledermatologist suspected skin cancer in 20.1% (2214/11,005) of the lesions (nonmelanoma skin cancer [keratinocytic]: 1707/11,005, 15.5%; melanoma: 507/11,005, 4.6%).

The most common benign diagnosis was benign melanocytic naevus (2933/11,005, 26.7%); naevi were classified as atypical or dysplastic in 236 of these cases. Other diagnoses included benign keratosis (2576/11,005, 23.4%), premalignant skin lesions (1132/11,005, 10.3%), other benign lesions (707/11,005, 6.4%), vascular lesions (325/11,005, 3%), inflammatory conditions (187/11,005, 1.7%), dermatofibromas (187/11,005, 1.7%), and nail abnormalities (125/11,005, 1.1%).

There were 291 skin lesions with no specific diagnosis (did not require further assessment), 206 nondiagnostic skin lesions (required face-to-face outpatient clinic appointments), 96 lesions that had resolved prior to imaging, 18 collision lesions with more than 1 diagnosis, and 8 treatment-related lesions.

The diagnosing dermatologist recommended excision for 744 lesions due to a high suspicion (523/744, 70.3%) or mild suspicion (221/744, 29.7%) of melanoma. Of the 523 with a high suspicion of melanoma, 320 were confirmed based on histology (melanoma in situ: n=209; invasive melanoma: n=111). The PPV of melanoma and melanoma in situ in this study was 61.1% (320 true positives and 203 false positives); in other words, there was 61.1% diagnostic agreement between the teledermatologist and the histopathology. Among the 744 excised lesions, there were 367 (49.3%) confirmed melanomas; 243 (243/367, 66.2%) were melanoma in situ and 124 (124/367, 33.8%) were invasive melanoma. The ratio of melanoma in situ to invasive melanoma (243:124) was 1.96. The ratio of the total number of excisions to the number with a histological diagnosis of melanoma or melanoma in situ was the NNT (2.02).

### Quality Standards

Our teledermoscopy service met 6 of the 8 British Primary Care Commissioning’s Quality Standards for Teledermatology [12].

There were clear guidelines on referral pathways for general practitioners, and only patients with skin lesions suspicious of cancer were referred. Informed consent was obtained from all patients. All images were taken by competent staff, and diagnoses were made by dermatologists who had experience in teledermatology.

The median waiting time for patients with suspected melanoma has exceeded the 2-week waiting time target. The Quality Standards for Teledermatology recommend that 1 audit and 1 patient survey be conducted every 12 months. We have conducted several partial audits over the years (some are included in this paper) but only conducted a single, limited patient survey [11].

## Discussion

The teledermoscopy service was able to deliver more efficient health care and improved access to specialist diagnoses.

### Principal Results

We have shown that community-based teledermoscopy clinics can reduce the need for dermatology outpatient appointments. The preponderance of women who attended the clinic (4087/6479, 63.1%) corresponds with the greater utilization of primary health care services by women [13]. The highest rate of melanoma was found in men and in the 65 to 74 years age group. Our figures confirm the prevalence of melanoma in fair-skinned individuals who are predominantly of European ancestry. The high risk of melanoma in this population can be explained through genetic predisposition and risk behavior [14]. Our sex and age group findings were comparable to the New Zealand Cancer Registry data from 2015 to 2017, in which a melanoma diagnosis is most common in men and in the 70 to 74 years age group. Further, 89.5% (5800/6479) of patients who attended clinic self-identified as New Zealand European in our study; however, Statistics New Zealand reported that the New Zealand population has a smaller proportion of New Zealand European individuals (74%) [15].

Seasonal variations in visits showed peaks in referrals and imaging at the end of summer and may have been due to (1) high lesion visibility during summer due to light clothing and (2) high skin cancer awareness during summer months. This is consistent with melanoma incidence data for New South Wales, Australia [16].

In terms of efficiency, the important outcomes were the reductions in waiting times and the streamlining of referrals. The median waiting time for patients with suspected melanoma exceeded the 2-week waiting time target. This may have been due to delays in the receipt and triage of general practitioner referrals, delays in sending out appointment letters, or high patient workloads. Most patients (233/306, 76.1%) were discharged or referred back to primary care.

The PPV of 61.1% and NNT of 2.02 show strong diagnostic concordance for melanoma. The melanoma in situ to invasive melanoma ratio of 1.96 indicates a high sensitivity for the diagnosis of melanoma. The high percentage of benign lesions diagnosed is encouraging, as without an expert opinion, many of these may have been subjected to unnecessary diagnostic procedures. The clinical photographs that were taken by a trained nurse (ie, a melanographer) using standardized equipment were of consistently high quality, allowing confident diagnoses to be made by our experienced dermatologists.

The results above are important in terms of improving health care access and delivery for the wider population. Our recommendations for current services include increasing the number of locations for imaging clinics, recruiting nurses with an appropriate level of training, and developing fast-tracked referral guidelines for high-risk individuals.

### Comparison With Prior Work

The diagnostic classifications in this study were comparable to those of other large teledermoscopy services. Mehrtens et al [8] reported benign naevus, seborrheic keratosis, and keratinocytic cancer in 25%, 22%, and 23% of 40,201 patients who attended a teledermoscopy service in the United Kingdom, respectively. Moreno-Ramirez et al [9] reported benign naevus, seborrheic keratosis, and keratinocytic cancer in 23%, 23.8%, and 10.4% of 34,553 patients who attended a teledermoscopy service in Spain, respectively.

### Limitations

Through our service, imaging is offered in 3 locations, so patients must travel beyond their primary care facility to access the service. The main concern has been prolonged delays prior to imaging.

Referrals to the service described herein decreased from mid-2017 onward, that is, after the introduction of an electronic referral pathway for suspected skin cancer that encourages referrers to attach their own clinical and dermoscopic images.

We have not undertaken a formal retrospective review of the lesions that were diagnosed as benign via teledermoscopy, so we cannot report the false-negative rate. However, the high percentage of benign lesions diagnosed in this study is encouraging. A systematic review of the data for the Auckland service reported a negative predictive value of 96%, with 2 false-negative diagnoses of melanoma [10].

### Conclusions

The teledermoscopy imaging service we have described has provided accurate diagnoses, thereby minimizing unnecessary visits to outpatient clinics, so that patients with confirmed skin cancer can be prioritized for surgery.

## Abbreviations

NNT: number needed to treat
PPV: positive predictive value
